# Supplementary material for: Lycium barbarum (Wolfberry) Branches and Leaves Enhance the Growth Performance and Improve the Rumen Microbiota in Hu Sheep
Source: Animals (Basel). 2024 May 29;14(11):1610. doi: 10.3390/ani14111610 (PMC11171408; doi:10.3390/ani14111610)
Supplement: Supplementary file 1 [file animals-14-01610-s001.zip › animals-2982614-supplementary.pdf]

Table S1. Effects of different proportions of LBL on Alpha diversity of rumen flora in Hu sheep (n=5).

Items	CON	LBL1	LBL2	LBL3	LBL4	SEM	*P*-value
ACE	1202.28	1422.21	1580.86	1555.57	1640.14	69.383	0.079
Chao1	1216.65	1442.20	1602.31	1574.27	1652.92	69.707	0.081
Simpson	0.99	0.99	0.99	0.99	0.99	0.000	0.188
Shannon	8.55	8.82	8.96	8.83	9.01	0.072	0.077
Coverage	0.99	0.99	0.99	0.99	0.99	0.000	0.115

Table S2. Effects of different proportions of LBL on the level of rumen flora in Hu sheep (n=5).

Items	CON	LBL1	LBL2	LBL3	LBL4	SEM	*P*-value
Phylum level							
Firmicutes	49.01	47.9	44.32	47.14	46.67	1.312	0.857
Bacteroides	35.64	40.65	42.45	33.74	42.05	2.595	0.809
Spirochaetota	1.70^{b}	2.02^{b}	3.88^{a}	3.07^{ab}	2.82^{ab}	0.276	0.045
Fibrobacterota	1.41	2.49	3.29	2.95	2.44	0.318	0.420
Patescibacteria	4.00	0.97	0.48	3.07	0.58	0.811	0.562
Verrucomicrobia	1.79	1.36	1.33	1.63	1.87	0.139	0.660
Proteobacteria	1.22	0.87	1.11	2.15	1.12	0.172	0.218
Actinobacteria	1.61	0.92	0.59	1.74	0.29	0.385	0.755
Cyanobacteria	2.15	0.21	0.52	1.80	0.32	0.448	0.551
Desulfobacterota	0.43	0.55	0.57	0.49	0.72	0.038	0.143
Others	1.02	1.30	1.45	2.19	1.10	0.148	0.114
Unassigned	0.01	0.75	0.01	0.06	0.00	0.155	0.483
Genus level							
uncultured_rumen_bacterium	12.52	9.82	11.024	11.44	11.40	0.544	0.658
Prevotella	9.78	12.22	9.54	10.26	11.37	1.281	0.966
Rikenellaceae_RC9_gut_group	6.37	6.40	7.83	5.39	6.63	0.619	0.850
unclassified_F082	4.18	4.18	7.54	3.04	5.40	0.616	0.194
Succiniclasticum	3.17	3.45	3.97	2.38	4.72	0.336	0.284
Saccharofermentans	4.13	3.16	3.56	2.29	3.30	0.242	0.226
unclassified_Lachnospiraceae	2.51	3.90	2.51	3.07	3.45	0.222	0.184
Christensenellaceae_R_7_group	3.05	2.03	1.87	4.79	2.32	0.588	0.601
Treponema	1.65	1.90	3.77	2.98	2.79	0.273	0.070
Fibrobacter	1.40	2.49	3.28	2.94	2.44	0.317	0.420
Others	51.22	49.70	45.09	51.36	46.19	1.783	0.749
Unassigned	0.01	0.75	0.01	0.06	0.00	0.155	0.483
